# From Strain to Substance: A Novel Approach to Red Yeast Rice Quality Assessment and Chromatographic Fingerprinting

**DOI:** 10.3390/jof12100734

**Published:** 2026-10-01

**Authors:** Fang Li, Shan Yang, Yue Zhang, Yuting Ye, Wenjuan Yang, Guangyuan Yang, Weifeng Huang, Daqiang Wu

**Affiliations:** 1 College of Traditional Chinese Medicine, Bozhou University, Bozhou 236800, China; lfahuang@163.com (F.L.);; 2 Department of Pathogenic Biology and Immunology, College of Integrated Chinese and Western Medicine, Anhui University of Chinese Medicine, 350 Longzihu Road, Hefei 230012, China; ys2003@stu.ahtcm.edu.cn (S.Y.);; 3 Anhui Dechang Pharmaceutical Co., Ltd., Bozhou 236800, China

**Keywords:** red yeast rice, ITS sequence, monacolin K, ergosterol, γ-aminobutyric acid, fingerprint spectrum

## Abstract

Red yeast rice has notable lipid-lowering efficacy, but strain diversity among *Monascus* sources complicates quality evaluation. To address this issue, a systematic analytical framework integrating morphological and molecular identification of *Monascus* strains, quantitative detection of key bioactive components, and chromatographic fingerprinting was established. Five *Monascus* species were identified by morphology and ITS sequencing. Quantitative analysis revealed wide concentration ranges for acid Monacolin K (0.15–23.29 mg/g), lactone Monacolin K (0.03–14.22 mg/g), ergosterol (0.94–22.79 mg/g), guanosine (10.35–852.19 μg/g), adenosine (0.06–147.96 μg/g), γ-aminobutyric acid (2.76–1217.43 μg/g), and β-glucan (0.08–4.73 mg/g). HPLC fingerprinting established eight characteristic common peaks with similarity coefficients above 0.802, and fingerprint-based analysis revealed considerable differences in bioactive component contents among species: *M. sanguineus* and *M. ruber* had the lowest levels, *M. fumeus* and *M. fuliginosus* the highest, and *M. purpureus* intermediate. This multi-dimensional framework integrating strain identification and component quantification provides a reliable tool for comprehensive quality assessment of red yeast rice.

## 1. Introduction

Red yeast rice, a natural green food derived from red yeast (mainly *Monascus purpureus*) fermented on rice, is recognized for its cardiovascular health-regulating and immune-boosting properties [1]. Red yeast pigment, safe and natural microbial colorants, and red yeast-fermented rice play significant roles in the food industry [2]. As a traditional edible filamentous fungus with over a millennium of use in China, red yeast can be fermented on rice under suitable conditions to produce red yeast rice [3]. The preparation method for red yeast rice is as follows: choose high-quality rice; after washing, soaking (about 4 h) and steaming (about 30 min until ripe and non-adhesive), cool to about 35 °C, inoculate *M. purpureus* spores (accounting for 0.5–1% of the weight of rice), and stir thoroughly. The pigment was produced by incubation at 32 °C for 48 h in the early stage, maintained at 28 °C for 120 h in the middle stage, and the active ingredients were accumulated by fermentation at 25 °C for 72 h in the late stage, during which regular turning was required. After fermentation, the yeast was dried at a low temperature of 45 °C until moisture was ≤12%, sifted to remove mycelia residues, and finally red yeast rice was produced. Reported biological activities of red yeast rice, including hypolipidemic, antihypertensive, and antimicrobial effects, have garnered increasing research interest [4]. The metabolic profile of red yeast rice can be tailored by varying fermentation substrates, incorporating exogenous compounds, or co-culturing with other microorganisms to achieve directed metabolite enrichment [5].

Current identification methods for red yeast rice primarily rely on morphological and molecular biological approaches. Traditional morphological identification, however, is limited by subjectivity, requiring significant expertise and experience, and is prone to misclassification, particularly for closely related species [6,7]. Molecular identification via polymerase chain reaction (PCR) amplification and sequencing of conserved homologous sequences (500–800 base pairs, bp), followed by multiple sequence alignment and phylogenetic analysis, enables rapid, accurate, and automated species identification [8]. Currently, red yeast rice on the market is produced by a variety of *Monascus* species, but the relationship between the *Monascus* species and the effective components of red yeast rice has not been fully elucidated.

High-Performance liquid chromatography (HPLC) detection is conducive to the efficient analysis of extracting active ingredients, optimizing the process and improving the extraction efficiency. For instance, some studies [9] quantitatively analyzed lovastatin analogues in red yeast rice using high-performance liquid chromatography, providing a quality benchmark for functionally fermented red yeast rice foods [10]. Studies have found that *Monascus purpureus* can assimilate cholesterol to a certain extent in vitro and reduce cholesterol content [11]. It is indicated that there are considerable differences in the contents of adenosine, adenine and lovastatin. Moreover, the nucleoside components in red yeast rice are involved in DNA metabolism and have various biological activities, including antitumor, anti-inflammatory, lipid-lowering and antiviral effects [12]. The quality evaluation of red yeast rice can be identified through the content of the above-mentioned components.

Therefore, we aim to analyze and quantify active components in red yeast rice using HPLC and establish a standardized technical system for component identification. This study will extend HPLC analysis to include ergosterol, uracil, cytidine, hypoxanthine, adenine, inosine, guanosine, thymidine, adenosine, and γ-aminobutyric acid, aiming to establish the reference framework for product quality evaluation and detection of red yeast rice, and to analyze and determine the effects of different *Monascus* fungi on the yield of active ingredients.

## 2. Materials and Methods

### 2.1. Drugs and Reagents

The reference standards used in this study included lovastatin (Batch No.: 100600-202006, purity: 99.6%) and guanosine (Batch No.: 111977-202202, purity: ≥93.6%), both obtained from the National Institutes for Food and Drug Control (NIFDC) in China. Additionally, ergosterol (Batch No.: PS010695, purity: ≥98.0%), uracil (Batch No.: PS020117, purity: ≥98.0%), cytidine (Batch No.: PS020733, purity: ≥98.0%), hypoxanthine (Batch No.: PS020061, purity: ≥98.0%), adenine (Batch No.: PS020156, purity: ≥98.0%), inosine (Batch No.: PS010437, purity: ≥98.0%), thymidine (Batch No.: PS020196, purity: ≥98.0%), adenosine (Batch No.: PS020496, purity: ≥98.5%), and γ-aminobutyric acid (Batch No.: PS2231204-10, purity: ≥98.0%) were utilized. A total of 13 batches of red yeast rice samples were employed in this experiment, sourced from Gutian County, Fujian Province; Huzhou City, Zhejiang Province; Wuhan City, Hubei Province; and Anhui Dechang Pharmaceutical Co., Ltd. (Bozhou, China). The samples were coded as F1-F11, ZJ1-ZJ2, respectively. The specific origins of these samples are detailed in Table 1. Another 14 samples of red yeast rice (Table S1) were also used to validate the HPLC fingerprints framework.

### 2.2. Isolation and Morphology of Red Yeast Rice Strains

First, we weighed 1 g of red yeast rice powder and added it to 90 mL of sterile physiological saline containing glass beads. We shook the mixture for 10 min and performed 10-fold serial dilutions. We pipetted 0.1 mL of the dilution and spread it onto an isolation Potato Dextrose Aga (PDA) medium. We incubated the plates at 30 °C for 7 days. From each sample, we picked at least five single colonies and transferred them to fresh isolation medium for cultivation. After three rounds of isolation and purification, we obtained relatively pure strains. We streaked the spores of the selected strains onto morphological identification Malt Extract Agar (MEA) medium plates, placed the plates in an incubator, and incubated them inverted at 30 °C. After 5–7 days under dark conditions, we observed the colony morphology and mycelial and spore morphology of each strain. We preliminarily identified the Monascus species based on morphology and pigment production. Initially, the colonies exhibited a pale orange hue, which deepened over time, presenting a velvety texture. The colonies displayed radial wrinkling with a characteristic four-concentric-ring color distribution. The central region appeared white and woolly, accompanied by the secretion of red droplets on the mycelial surface. This was succeeded by light red and dark red zones, with the outermost layer of light-yellow mycelium and the colony margins being relatively even. The mycelia were septate and highly branched. The cleistothecia were spherical, measuring 30–50 μm in diameter, and ranged in color from orange to brown. The ascospores were nearly spherical to pear-shaped hyaline (colorless and transparent). The results are illustrated in Figure 1.

### 2.3. Identification of PCR Amplification Products by Agarose Gel Electrophoresis

For the amplification of the ITS gene by PCR, the primers ITS1 (5′-TCCGTAGGTGAACCTGCGG-3′) and ITS4 (5′-TCCTCCGCTTATTGATATGC-3′) were employed. The resulting PCR products were analyzed using 0.8% agarose gel electrophoresis to assess their size and specificity. Purified PCR products were subsequently sent to Sangon Biotech (Shanghai) Co., Ltd. (Shanghai, China) for sequencing. The obtained sequences were then subjected to BLASTN (version: 2.17.0) alignment against the GenBank database to determine the species information.

### 2.4. Determination of Monacolin K Content and Its Analytical Methodology

In this study, chromatographic analysis was conducted using a WondaSil C18 Superb 5 μm column with a mobile phase of acetonitrile (A) and 0.1% phosphoric acid (B) under isocratic elution (60% A from 1 to 23 min) at a column temperature of 30 °C, detection wavelength of 238 nm, flow rate of 1.0 mL/min, and injection volume of 10 μL by LC-2030Cplus Shimadzu High-Performance Liquid Chromatograph (Shimadzu Corporation, Kyoto, Japan). For test solution preparation, a precisely weighed 1.0 g portion of red yeast rice powder was extracted with 15 mL of 75% ethanol via sonication for 30 min, cooled, filtered, and the filtrate was passed through a 0.45 μm membrane filter. The contents of acid and lactone Monacolin K (also named as lovastain) were quantified using this method. Reference solutions were prepared by dissolving lactone lovastatin reference standard in methanol (for lactone-form) or, for acid-form Monacolin K, by dissolving lactone lovastatin in methanol, adding 25 mL of 0.2 mol/L NaOH solution, sonicating for 30 min, neutralizing with 25 mL of 0.2 mol/L HCl solution, and adjusting the volume to the mark with purified water. Both reference solutions were injected under the specified conditions to construct standard curves. Precision was evaluated by six consecutive injections of 10 μL of acid and lactone Monacolin K reference solutions (25 μg/mL), with peak areas recorded for analysis.

### 2.5. Determination of Ergosterol Content

The chromatographic conditions employed in this determination are as follows: a Cosmosil 5C18-MS-II column (4.6 mm × 250 mm) was utilized, with methanol (A) as the mobile phase under isocratic elution (100% A from 1 to 16 min). The column temperature was maintained at 30 °C, the detection wavelength was set at 284 nm, the flow rate was 1.0 mL/min, and the injection volume was 20 μL. For the preparation of the test sample solution, 1.0 g of red yeast rice powder was accurately weighed and added to 20 mL of a 25% alkaline–ethanol solution (prepared by dissolving 25 g of NaOH in 40 mL of water and diluting to 100 mL with absolute ethanol). The mixture was saponified in a water bath at 85–90 °C for 1 h, followed by adding 2 mL of absolute ethanol and further saponification for an additional 0.5 h. After cooling, 10 mL of ether was added, and the mixture was shaken for 30 s and allowed to stand for 30 min. The ether layer was separated, washed with water until neutrality, evaporated to dryness, and then diluted to 25 mL with absolute ethanol. The solution was passed through a 0.45 μm membrane filter to obtain the test sample solution. The content of ergosterol was determined using the method above. To prepare the reference standard solution, the ergosterol reference standard was accurately weighed and dissolved in methanol to obtain the reference standard solution. The ergosterol reference standard was accurately weighed and dissolved in methanol to prepare standard solutions of different concentrations to establish the standard curve. These solutions were injected and analyzed under the chromatographic conditions, and the peak areas were recorded to plot the standard curve. For the precision experiment, 10 μL of the ergosterol reference standard solution with a 97.5 μg/mL concentration was aspirated and injected six times consecutively under the chromatographic conditions. The peak area data were recorded.

### 2.6. Determination of Nucleoside Components’ Content

Chromatographic analysis was conducted using a Cosmosil 5C18-MS-II column (4.6 mm × 250 mm) with methanol (A)-ultrapure water (B) mobile phase under gradient elution (2–5% A, 0–10 min; 5–15% A, 10–15 min; 15–17% A, 15–22 min; 17–20% A, 22–26 min; 20–2% A, 26–37 min), at 30 °C, 260 nm, 1.0 mL/min, and 20 μL injection volume. For the test solution, 1.0 g of red yeast rice powder was extracted with 10 mL ultrapure water via sonication (20 min), filtered, and passed through a 0.45 μm microporous organic membrane filter. Reference solutions were prepared by dissolving individual nucleoside standards in ultrapure water. A standard curve was established by diluting these references into six gradient-concentration mixed solutions, which were injected to record peak areas. Precision was evaluated by six consecutive injections of a mixed reference solution containing 10 μL aliquots of uracil (6.25 μg/mL), cytidine (8.5 μg/mL), hypoxanthine (11 μg/mL), adenine (8.75 μg/mL), inosine (28 μg/mL), guanosine (2.5 μg/mL), thymidine (3.75 μg/mL), and adenosine (1.5 μg/mL), with peak areas recorded for analysis.

### 2.7. Determination of GABA Content

The chromatographic conditions employed in this determination are as follows: a Cosmosil 5C18-MS-II column (4.6 mm × 250 mm) was utilized. The mobile phase consisted of acetonitrile (A) and 0.02 mol/L ammonium acetate (B), with isocratic elution (19% A from 0 to 56 min). The column temperature was maintained at 30 °C, the detection wavelength was set at 320 nm, the flow rate was 1.0 mL/min, and the injection volume was 20 μL. A total of 1.0 g of red yeast rice sample powder was accurately weighed and placed in a 15 mL centrifuge tube to prepare the test sample solution. Ten mL of 60% ethanol was added to dissolve the sample, followed by ultrasonic extraction for 20 min. The mixture was then centrifuged at 4000 r/min for 20 min. Precisely, 0.2 mL of the supernatant was aspirated and mixed with 0.8 mL of an OPA derivatization solution (prepared by dissolving 0.3 g of OPA in 15 mL of methanol, then adding 60 mL of 0.4 mol/L boric acid buffer (pH 10.2) and 0.9 mL of β-mercaptoethanol, and allowing the mixture to stand for 2 days). The solution was shaken for 5 s, allowed to stand for 2 min, and then passed through a 0.45 μm membrane filter to obtain the test sample solution. Within 3 min, 20 μL of the solution was injected and analyzed using the above method to calculate the γ-Aminobutyric Acid (GABA) content. An appropriate amount of GABA reference standard was accurately weighed and dissolved in 60% ethanol to prepare the reference standard solution. A GABA reference standard solution was precisely prepared to establish the standard curve. Then, 0.2 mL of this solution was mixed with the OPA derivatization solution to a final volume of 1.0 mL. After shaking and allowing the mixture to stand for 2 min, it was passed through a 0.45 μm membrane filter, and 20 μL was injected. The sample was analyzed under the chromatographic conditions, the peak area was recorded, and the standard curve was plotted. For the precision experiment, 10 μL of a GABA reference standard solution with a 100 μg/mL concentration was precisely aspirated and injected six times consecutively under the chromatographic conditions.

### 2.8. Determination of β-Glucan Content

Precisely pipette 1.0 mL of the β-glucan reference standard solution and add it to 4.0 mL of a Congo red solution (prepared by accurately weighing 0.3 g of Congo red, dissolving it in 0.1 mg/mL phosphate buffer at pH 8.0, and diluting to a final volume of 100 mL). Vortex the mixture to ensure thorough mixing, then allow it to stand at 25 °C for color development for 30 min. Measure the absorbance at 545 nm using a UV-1900i UV-Vis Spectrophotometer (Shimadzu Corporation, Kyoto, Japan). For the preparation of the test sample solution, accurately weigh 1.0 g of red yeast rice powder, add 25 mL of ultrapure water, seal the container, and perform ultrasonic extraction for 10 min. Heat the mixture in a water bath at 70 °C for 1 h. Take 1 mL of the subsequent filtrate, add 4 mL of a 3 mg/mL Congo red solution, allow it to stand for color development for 30 min, and then measure the absorbance according to the chromatographic method to calculate the β-glucan content. For the preparation of the reference standard solution, accurately weigh the β-glucan reference standard, dissolve it in ultrapure water, and heat it in a water bath at 70 °C for 60 min until complete dissolution is achieved. To establish the standard curve, precisely prepare β-glucan reference standard solutions. Pipette 1.0 mL of each reference standard solution into separate test tubes, add 4.0 mL of a 3 mg/mL Congo red solution, and vortex to mix well. Measure the absorbance of each concentration of the reference standard solution under the chromatographic conditions specified for β-glucan. Record the absorbance values and plot the standard curve. For the precision experiment, pipette the β-glucan reference standard solution with an 80 μg/mL concentration. Under the aforementioned chromatographic conditions, measure the absorbance six times consecutively.

### 2.9. Determination of the Citrinin Concentration

The sample is ground finely. A 2 g portion of the sample is accurately weighed, and 10 mL of 30% methanol is accurately added. The mixture is weighed, ultrasonicated (250 W, 40 kHz) for 30 min, made up to the original weight with 30% methanol, shaken well, and centrifuged at 3000 rpm for 5 min. The supernatant (1 mL) is accurately pipetted into a 25 mL volumetric flask, diluted to volume with PBS buffer (pH 7.0), shaken thoroughly, and filtered. The subsequent filtrate (10 mL) is accurately pipetted and passed through an immunoaffinity column pretreated with 2 mL PBS at a flow rate of less than 3 mL/min, and air is allowed to enter the column. The column is washed with 10 mL of 0.1% Tween PBS wash solution, and the washings are discarded. Air is allowed to enter the column to expel the washing solution. The column is eluted with an appropriate amount of methanol–0.1% phosphoric acid solution (70:30). The eluate is collected in a 2 mL volumetric flask, diluted to volume with the same solution, shaken well, filtered through a 0.22 μm microporous membrane, and the subsequent filtrate is collected.

The analytical column is packed with octadecylsilane-bonded silica gel (column length 100 mm, inner diameter 2.1 mm, particle size 1.6 µm). Mobile phase A is 0.2% formic acid solution, and mobile phase B is 0.2% formic acid in acetonitrile. Gradient elution is performed according to the table below. The flow rate is 0.3 mL/min, and the column temperature is 25 °C. A triple-quadrupole tandem mass spectrometer is used with electrospray ionization (ESI) in positive ion mode and multiple reaction monitoring (MRM). The citrinin reference standard is accurately weighed, and methanol is added to prepare a stock solution containing 1 µg per 1 mL. Serial dilutions are prepared and detected, and a standard curve is constructed. Separately, 5 µL of the above test solution is accurately injected into the HPLC-MS instrument. The peak area is measured, the amount equivalent to the reference standard in the test sample is read from the standard curve, and the citrinin concentration is calculated.

### 2.10. Establishment and Analysis of the Fingerprint Profile

The chromatographic conditions employed in this determination are as follows: a WondaSil C18 Superb 5 μm column was utilized. The mobile phase consisted of acetonitrile (A) and 0.05% phosphoric acid (B), with gradient elution (0–45 min, 25–84% A; 45–55 min, 84–25% A; 55–65 min, 25% A). The column temperature was maintained at 30 °C, the detection wavelength was set at 256 nm, the flow rate was 1.0 mL/min, and the injection volume was 20 μL. For the preparation of the test sample solution, 0.5 g of red yeast rice sample powder was accurately weighed and placed in a conical flask. A total of 25 mL of a 30% methanol solution was precisely added, and the flask was sealed. The solution was subjected to ultrasonic extraction for 90 min. After cooling the solution to room temperature, the filtrate was taken and passed through a 0.45 μm microporous organic membrane filter. Under the aforementioned chromatographic conditions, the prepared test sample solution was injected for analysis to obtain the fingerprint profile of the red yeast rice sample.

Additionally, a reference standard solution was prepared and injected under the chromatographic conditions to get the chromatogram of the lovastatin reference standard. For the precision experiment, 1.0 g of red yeast rice sample powder was accurately weighed, and the test sample solution was prepared according to the method above. Six consecutive injections were performed for analysis under chromatographic conditions.

## 3. Results

### 3.1. Morphological Characterization and Species Identification of Isolated Red Yeast Strains

Based on the comprehensive analysis of experimental data, including the cultural characteristics, microscopic morphological features of hyphae and spores (Figure 1), and ITS sequences of the submitted strains under the conditions of our laboratory, the identification results are as follows (Table 1): samples F1, F3, F5, F8, F9, F10, F11, ZJ1 and ZJ2 were identified as *Monascus purpureus* species; sample F2 was identified as *Monascus fumeus*; sample F4 was identified as *Monascus fuliginosus* species; sample F6 was identified as *Monascus ruber* species; and sample F7 was identified as *Monascus sanguineus* species. Thus, our selected red yeast rice samples are fermented by five *Monascus* fungi species.

### 3.2. Analysis of Monacolin K Content

Red yeast rice can metabolize various bioactive substances, with lovastatin being one of its functional components known for its hypotensive and hypolipidemic treatment effects. Lactone Monacolin K and acid Monacolin K are the main characteristic components of functional *Monascus* and lipid-lowering products based on functional *Monascus*. The total content of Monacolin K is one of the important indicators for the quality control of functional *Monascus* products. The acid Monacolin K exhibits more significant biological activity [13]. Currently, the detection of Monacolin K using HPLC is well-established. In this study, we analyzed a batch of red yeast samples from different sources using the method described in “2.4”. An HPLC method for determining Monacolin K in red yeast rice was established to investigate the contents of Monacolin K’s acid and lactone forms. The regression equation for the acid lovastatin was y = 1879.7x − 13,145 (R^2^ = 0.9998), with a linear range of 3.125–1600 μg/mL. For the lactone form, the regression equation was y = 41320x − 4196.5 (R^2^ = 0.9996), with a linear range of 3.125–200 μg/mL. Methodological validation demonstrated that the relative standard deviations (RSDs) of the peak areas for the acid and lactone forms of lovastatin were 1.75% and 0.13% (instrument precision), respectively, 1.66% and 1.92% (repeatability), and 0.76% and 1.12% (stability), indicating suitable method precision, repeatability, and stability. The spike recoveries were 98.52% and 97.32%, respectively. This method analyzed 13 batches of red yeast rice samples from different sources. The results showed that the content of the acid Monacolin K ranged from 0.15 to 23.29 mg/g, while the content of the lactone form ranged from 0.03 to 14.22 mg/g (Figure 2B). The experimental results indicate that the HPLC method established in this study exhibits high precision and good repeatability, making it suitable for accurately determining Monacolin K content in red yeast rice.

### 3.3. Analysis of Ergosterol Content

Ergosterol, also known as ergocalciferol precursor or simply ergosterol (here referring to the compound itself), is an essential vitamin-like substance produced during the fermentation process of red yeast rice. It also serves as a key component of the microbial cell membrane. As a precursor of vitamin D_2_, ergosterol can undergo structural transformation under ultraviolet irradiation to generate vitamin D_2_. Vitamin D_2_ plays crucial physiological roles in the human body, including regulating calcium and phosphorus metabolism and influencing cell proliferation and differentiation [10]. In this study, the content of ergosterol was determined using HPLC. The regression equation, calculated based on the peak area obtained using the method described in “2.5”, was y = 2340.4x − 15,261 (R^2^ = 0.9998), indicating a good linear relationship for ergosterol within the concentration range of 23.44–1500 μg/mL. The instrument precision test’s relative standard deviation (RSD) of the ergosterol peak area was 0.33%, demonstrating the instrument’s good precision. The RSD of the ergosterol peak area in the repeatability experiment was 1.70%.

In contrast, in the stability experiment, the RSD of the ergosterol peak area for the test sample solution stored at room temperature for 24 h was 0.05%, indicating suitable method repeatability and solution stability. The average recovery rate of ergosterol in the spike recovery experiment was 98.51%. This method was applied to determine the ergosterol content in 13 batches of red yeast rice samples from different sources, and the results showed that the ergosterol content ranged from 0.94 to 22.79 mg/g (Figure 3A). The experimental procedure involved preparing the test sample solution, injecting it for analysis, recording the peak area and chromatogram according to the method described in “2.5”, and performing quantitative analysis using the standard curve method (Figure 3B). The accuracy and reliability of the technique were further validated by precisely aspirating the ergosterol reference standard solution and preparing a blank solution for comparative determination.

### 3.4. Analysis of Nucleoside Components’ Content

In addition to lovastatin and ergosterol, red yeast rice contains nucleoside components, which play significant roles in nutrient and flavor. As a class of bioactive constituents in TCM, nucleosides have been extensively studied in the context of quality control for TCM and its prepared medication. This study recorded the peak areas according to the method described in “2.6”. Using the peak area values as the ordinate and the concentrations of each sample as the abscissa, regression equations were derived. These results indicate a good linear relationship between the concentrations of the respective components and their corresponding peak areas within the examined concentration ranges.

Six consecutive injections were performed under the conditions specified in “2.6”. After recording the peak areas, the RSDs of the peak areas for each reference standard were calculated to be 0.09%, 1.28%, 0.08%, 0.15%, 0.04%, 1.18%, 0.34%, and 0.50%, respectively, indicating good instrumental precision. An accurately weighed 1.0 g portion of red yeast rice sample powder was used to prepare the test sample solution, followed by experiments to assess repeatability, stability, and spike recovery. The experimental results showed that the RSDs of the peak areas for each component ranged from 1.53% to 1.95% (repeatability) and from 1.20% to 1.97% (stability). The average recoveries ranged from 97.87% to 98.43% (spike recovery), demonstrating that the method employed possessed good repeatability, stability, and spike recovery. Test sample solutions were prepared from 13 red yeast rice samples obtained from various sources. These solutions were injected and analyzed under the specified chromatographic conditions, with the chromatograms recorded, as shown in Figure 4B. Precisely aspirated 0.2 μL aliquots of different sample reference standard solutions were combined to prepare a mixed reference standard solution, and the peak areas were determined under the chromatographic conditions. Additionally, a blank solution was prepared by filtering ultrapure water through a 0.45 μm membrane and analyzed under the same chromatographic conditions, with the results presented in Figure 4. The contents of uracil, cytidine, hypoxanthine, adenine, inosine, guanosine, thymidine, and adenosine in the 13 batches of red yeast rice samples from different sources were calculated using the standard curve method, as follows: uracil (0.081–97.608 μg/g), cytidine (6.572–158.45 μg/g), hypoxanthine (2.832–111.851 μg/g), adenine (3.372–151.65 μg/g), inosine (0.01–12.837 μg/g), guanosine (10.353–852.194 μg/g), thymidine (1.902–62.31 μg/g), and adenosine (0.061–147.964 μg/g).

### 3.5. Analysis of GABA Content

Red yeast species also synthesize beneficial secondary metabolites for human health, such as GABA [14]. In this study, to further characterize different batches of red yeast rice, the content of GABA present in red yeast rice was determined using HPLC. The peak areas were recorded, and a standard curve was plotted according to the method described in “2.7”, yielding the regression equation y = 3726.2x − 4171.7 (R^2^ = 0.9992). This indicates a good linear relationship for GABA within the concentration range of 3.125–100 μg/mL. The GABA peak area’s RSD was 1.55%, demonstrating good instrumental precision. An accurately weighed 1.0 g portion of red yeast rice sample powder was subjected to repeatability, stability, and spike recovery experiments following the method outlined in “2.7”. The RSDs of the GABA peak area were measured to be 1.73% (repeatability) and 42.85% (stability, comparing results at 0 and 1 h), with an average recovery rate of 98.68% (spike recovery). These results suggest that the method exhibits good repeatability but relatively poor stability, necessitating immediate sample injection and analysis. Test sample solutions were prepared from 13 batches of red yeast rice samples obtained from various sources, injected, and analyzed, with the peak areas and chromatograms recorded (as shown in Figure 5). A GABA reference standard solution and a blank solution (60% ethanol + OPA derivatization reagent) were also prepared and analyzed. Using the standard curve method, the GABA content in each sample was calculated to range from 2.76 to 1217.43 μg/g.

### 3.6. Analysis of β-Glucan and Citrinin Content

During the fermentation of red yeast, various bioactive substances are produced, including β-glucan. β-glucan has been shown to reduce serum cholesterol levels by inhibiting intestinal absorption of dietary fats and cholesterol, promoting bile acid excretion, and accelerating the conversion of cholesterol to bile acids. Additionally, it exhibits potential anticancer and antitumor bioactivities. In this study, the β-glucan content in different batches of red yeast rice samples was quantified using UV-Vis. Absorbance measurements of β-glucan standard solutions were performed according to the method outlined in “2.8”, with blank absorbance values calibrated to zero using ultrapure water and Congo red solution, demonstrating reasonable method specificity. The standard curve regression equation was determined to be y = 0.0041x − 0.0331 (R^2^ = 0.9925), with a linear range of 40–200 μg/mL, indicating a robust linear relationship for β-glucan within this concentration interval. The absorbance of an 80 μg/mL β-glucan solution was measured six times consecutively, yielding an RSD of 0.77%, confirming excellent instrumental precision. Six replicate preparations of red yeast rice test sample solutions were analyzed, resulting in an absorbance RSD of 1.16%, underscoring the method’s good repeatability. When test solutions were assayed at various time points over 24 h, the absorbance RSD increased to 8.98%, suggesting that measurements should be conducted promptly after sample preparation to ensure accuracy. The spike recovery test yielded an average recovery rate of 98.62%. Finally, using the standard curve method, the β-glucan content in 13 batches of red yeast rice samples from diverse sources ranged from 0.080 to 4.734 mg/g.

Because citrinin is an important potential toxic metabolite of red yeast rice products, we also measured the citrinin content of 13 samples. The results (Table S2) showed that citrinin was detectable in 3 of the 13 samples, namely F3, F5, and F11, at concentrations of 140, 605, and 12,451 ng/mL, respectively. Therefore, the detection of citrinin is of great significance for the compositional and quality testing of red yeast rice.

### 3.7. Establishment and Analysis of Fingerprint Profiles

Fingerprint profiles can reflect variations in chemical constituents’ types and relative abundances in TCMs or pharmaceutical preparations, enabling comprehensive quality evaluation. To establish a standardized identification system for red yeast rice-derived products, this study conducted fingerprint analysis of red yeast rice samples from diverse sources. Following the method outlined in “2.10”, a consensus chromatogram of red yeast rice samples was generated using the Similarity Evaluation System for Chromatographic Fingerprints of TCMs [15], identifying eight typical peaks in the HPLC fingerprint. Peak 8 was confirmed as Monacolin K through comparison with a reference standard. Using peak eight as the reference, the relative peak area RSDs ranged from 68.49% to 94.59%, indicating substantial variability in constituent concentrations among red yeast rice samples (Figure 6A). In precision experiments, the RSDs of relative peak areas for all eight typical peaks were below 2.81%, demonstrating excellent instrumental precision. Six replicate preparations of test sample solutions yielded RSDs of relative peak areas below 2.89% for all eight peaks, confirming suitable method repeatability. When test solutions were stored at room temperature for 24 h, the RSD of peak areas for the eight typical peaks was 2.14%, indicating satisfactory solution stability. Similarity evaluations of in total 27 batches of red yeast rice samples, which contained the 13 above samples and the additional 14 samples, revealed similarity values above 0.802, suggesting consistent chemical profiles across batches (Figure 6B).

To sum up, red yeast rice is a component in the diet of some parts of Asia and is also used as a traditional Chinese medicine. Its main active ingredient is Monaclin K, and its lactone form is chemically similar to lovastatin. Therefore, it is a natural inhibitor of 3-hydroxy-3-methyl-glutaryl coenzyme A reductase (HMGCoA). HMGCoA is the rate-limiting enzyme for endogenous cholesterol production. Importantly, the intake of red yeast rice can reduce the circulating concentration of low-density lipoprotein particles that cause atherosclerosis. Studies have shown that this is significantly associated with a reduced risk of cardiovascular events, increasing the possibility of the role of red yeast rice preparations in preventing cardiovascular diseases. Like natural products, RYR contains a wide range of chemical components, and Monacolin K may contribute to lipid-lowering effects to varying degrees. The Monacolin K content per gram of genuine RYR samples ranges from 2.0 mg to 2.3 mg. Among the three batches of samples listed in this article, the content of Monacolin K in samples such as F1-F5, F9, and ZJ1 all exceeded that of genuine RYR samples. It can be seen that the content of red yeast rice produced in Fujian exceeds the industry standard for functional red yeast rice (powder) by 1.37 to 2.232 times. It helps to increase the exploration of red yeast rice strain resources, and enhances the quality of functional red yeast rice foods and their value in lipid-lowering and health preservation.

### 3.8. Comparative Analysis of the Effective Components of Red Yeast Rice from Different Species Sources

Based on the fingerprinting method for active components of *Monascus* species established in this study, we analyzed the contents of bioactive compounds and nucleotide components in thirteen samples, which are detailed in Table S3. The comparative results of bioactive compounds revealed considerable differences among the five *Monascus* species represented by these thirteen samples (Figure 7). Overall, the species classified as color-producing strains, *M. sanguineus* and *M. ruber*, exhibited the lowest levels of bioactive compounds. Notably, acid Monacolin K and lactone lovastatin were completely undetectable in *M. sanguineus* (Figure 7A). In contrast, *M. fumeus* and *M. fuliginosus* displayed generally higher levels of bioactive compounds, with *M. fuliginosus* showing the highest overall content. *M. purpureus*, which is currently the most commercially prevalent species, presented an intermediate level of bioactive compounds (Figure 7A). In the comparative analysis of nucleotide components (Figure 7B), we observed a similar pattern of interspecific variation. Overall, *M. ruber* had the lowest nucleotide content, while *M. fumeus* showed the highest. *M. purpureus* again exhibited an intermediate level. These findings indicate that nucleotide production also varies significantly following rice fermentation by different *Monascus* species. The results from this section demonstrate substantial differences in the levels of bioactive active ingredients and nutritional nucleotide components among *Monascus*-fermented food products derived from different fungal strains. The analytical method established in this study enables precise and efficient identification and quantification of these components.

## 4. Discussion

Here, we first determined the species of isolated *Monascus* strains. Then, we systematically optimized analytical methods for quantifying multiple bioactive components (Monacolin K, ergosterol, nucleosides, GABA) in red yeast rice samples. Seventy-five percent ethanol was chosen for lovastatin extraction due to its higher efficiency; saponification extraction was selected for ergosterol based on yield and chromatographic performance; mobile phase B (methanol–ultrapure water) was used for nucleoside quantification for its superior HPLC performance; and OPA derivatization was applied for accurate GABA quantification. For β-glucan, the 3 mg/mL Congo red solution was the optimal chromogenic agent. These optimized methods offer a reliable scientific basis for red yeast rice production quality control and compositional analysis.

In the quantification of nucleosides in red yeast rice samples, the inherent polarity of glycosidic components poses a challenge for mobile phase selection in reversed-phase high-performance liquid chromatography (RP-HPLC). To address this, a systematic evaluation was conducted comparing two mobile phases: mobile phase A (acetonitrile: potassium dihydrogen phosphate buffer) and mobile phase B (methanol: ultrapure water). Key chromatographic parameters such as retention time, peak symmetry, and resolution were thoroughly assessed. The results showed that mobile phase B delivered superior performance. It provided better resolution, symmetrical peaks with negligible tailing, and retention times that were ideal for separating nucleosides.

Through systematic comparative experiments and comprehensive evaluations of extraction solvents, methodologies, and chromatographic conditions, we established optimal protocols for extracting and quantifying lovastatin, ergosterol, nucleosides, and GABA from red yeast rice samples [16]. For GABA determination, given its specific chemical structure lacking a strong chromophore, pre-column derivatization with OPA reagent was necessary to enable sensitive ultraviolet fluorescence detection [17,18]. To ensure accurate quantification, both the reference standard and sample test solutions were derivatized. Moreover, considering the instability of OPA derivatives [17,19], all derivatized solutions were injected within a strict three-minute window after reagent addition. This approach guaranteed analytical precision and reproducibility [18]. These optimized procedures lay a solid scientific foundation for quality control and detailed compositional analysis of Monascus-derived products.

In the Congo red binding assay for β-glucan quantification, the concentration of the chromogenic agent (Congo red) is a crucial factor affecting absorbance stability [20]. To investigate this, we conducted experimental comparisons using Congo red solutions at two concentrations: 3 mg/mL and 1 mg/mL. We added the same concentration of a standardized β-glucan solution to both Congo red solutions and measured the absorbance under strictly controlled conditions, ensuring consistent instrument parameters and β-glucan concentration throughout [21]. The results showed that the 3 mg/mL Congo red solution provided significantly more stable absorbance values with minimal signal fluctuations compared to the 1 mg/mL solution [20]. Given this enhanced stability, we selected the 3 mg/mL Congo red solution as the chromogenic agent for our β-glucan quantification protocol [21].

Currently, there is a wide variety of red yeast rice products available on the market, but their quality and safety raise significant concerns. The most widely recognized species in the market is *M. purpureus*, which demonstrates effective lipid-lowering activity with minimal side effects. The comprehensive method established in this study for identifying active components confirmed that *M. sanguineus* and *M. ruber* contain the lowest levels of active ingredients, particularly Monacolin K, which is nearly undetectable. In contrast, *M. fumeus* and *M. fuliginosus* exhibit the highest overall content of active components, with Monacolin K levels reaching the maximum. This might pose a risk for consumers, as it becomes difficult to control appropriate intake, potentially leading to adverse reactions due to overdosing. *M. purpureus* presents an intermediate profile, possibly providing effective lipid-lowering effects with moderate consumption. Therefore, the findings of this study shed further light on the underlying reasons for the differences in active component composition and quality among red yeast rice products fermented by different *Monascus* species. The differences in efficacy of red yeast rice fermented by different *Monascus* strains, caused by variations in active ingredients, warrant further in vivo efficacy studies for elucidation.

However, the large number of samples, with diverse geographical origins, cultivation, and processing methods, posed challenges in data management and analysis, potentially increasing variability and hindering pattern identification. This diversity might also have affected method stability, and the high sample volume increased validation time and resource demands, limiting its thoroughness.

Future research should carefully select samples to balance comprehensiveness and manageability. Efforts should focus on enhancing sensitivity through advanced sample preparation and detectors, conducting rigorous validation, establishing bioactivity-composition links via pharmacological studies, and transferring optimized methods to industry with standardized procedures and large-scale trials. This will ensure product quality, safety, and efficacy, promoting standardized industrial and clinical use of red yeast rice.

In summary, this study established a technical framework for red yeast rice component identification through systematic experiments and rigorous analysis. The main components were isolated, identified, and quantified using modern techniques such as HPLC, gene sequencing, and UV-visible spectrophotometry. The methods showed excellent precision, repeatability, stability, and accuracy, aiding in quality control, efficacy evaluation, and safety assessment. Overall, our study provides a solid technical foundation and scientific evidence for further research and industrial applications of red yeast rice.

## Figures and Tables

**Figure 1:**
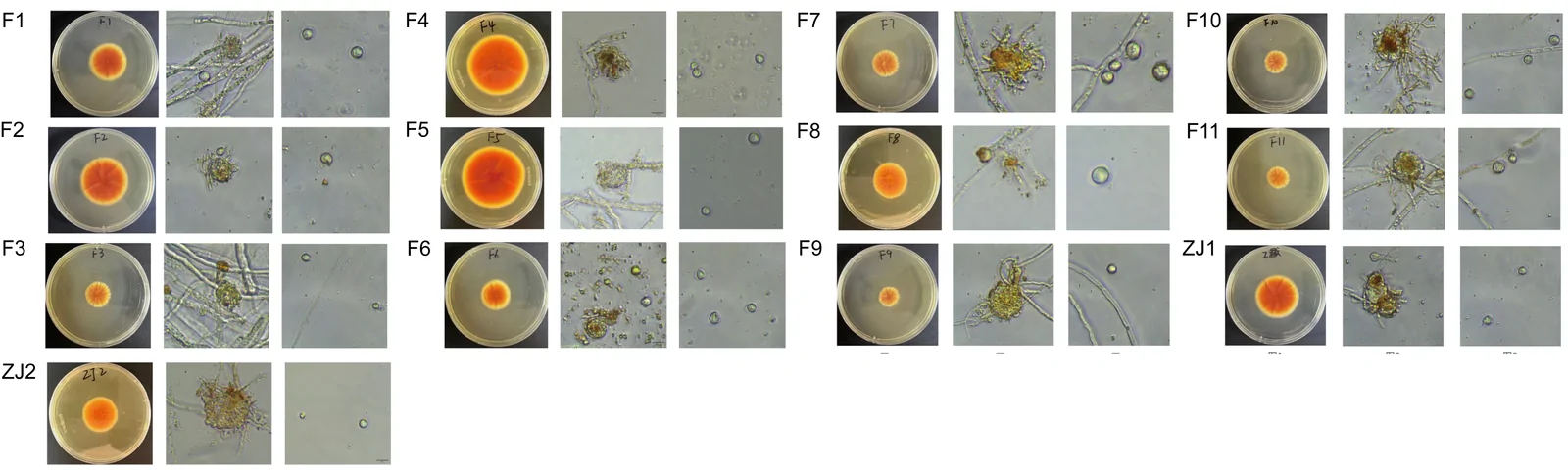
Cultural and microscopic characteristics of different red yeast rice samples. The colonies were light orange and then darkened, becoming velvety. The colonies are radially folded. The color of the colonies is distributed in four concentric circles. In the middle, there are white downy and red droplets secreted on the surface of the mycelium, followed by light red and dark red. The outermost layer is light-yellow mycelium. The edges are relatively neat, as can be seen from the left picture. Hyphae are septate and multibranched. The closed capsule shell is spherical, 30 to 50 um in diameter, and can be seen in the middle panel.

**Figure 2:**
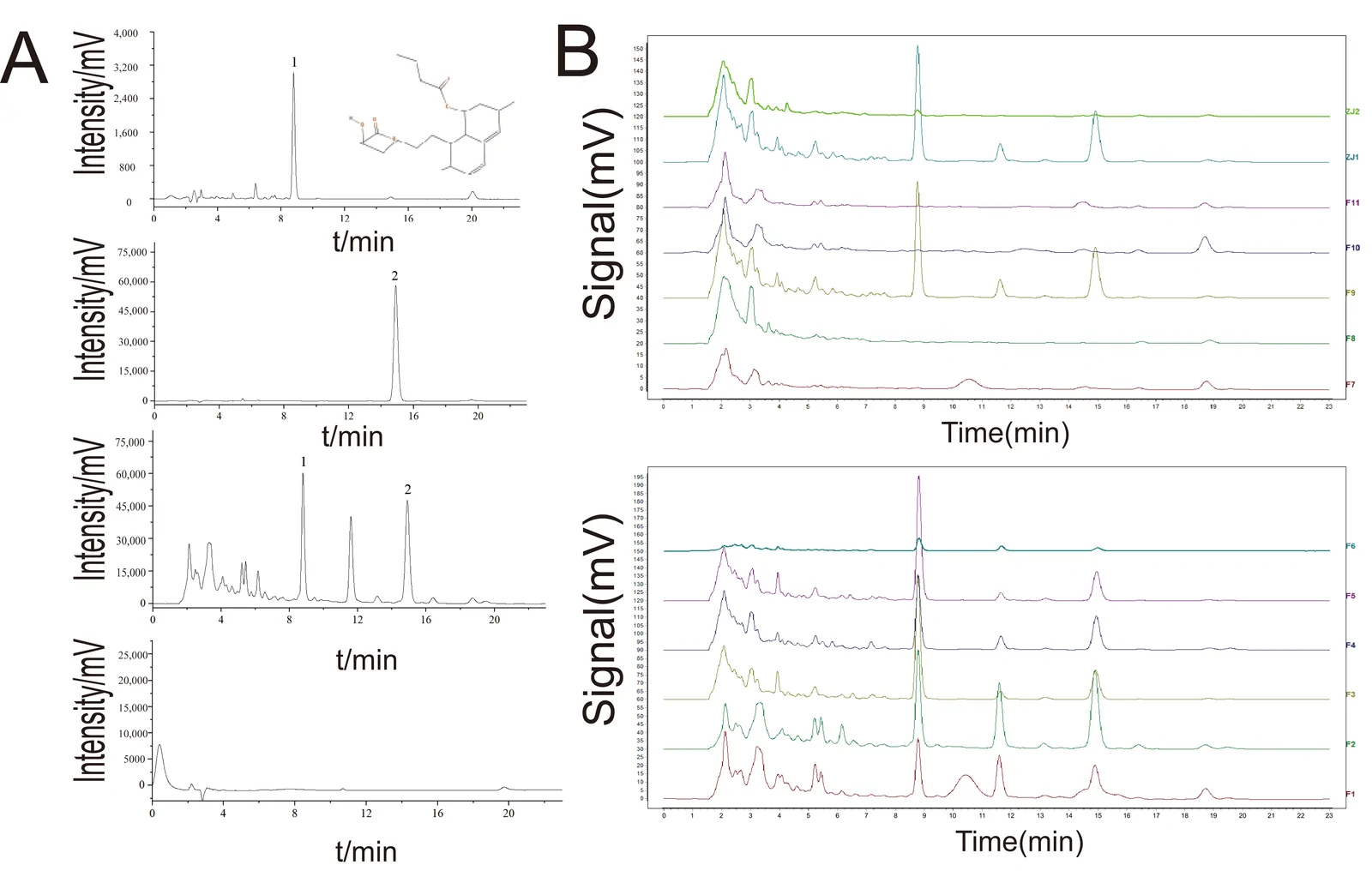
Monacolin K content determination chart. (**A**) Exclusive chromatogram of Monacolin K in red yeast rice samples from different sources. (**B**) Chromatograms of acid lovastatin and lactone Monacolin K in red yeast rice samples from different sources. 1 represents acid Monacolin K; 2 represents lactone Monacolin K. The curves in different colors represent the test results of different samples.

**Figure 3:**
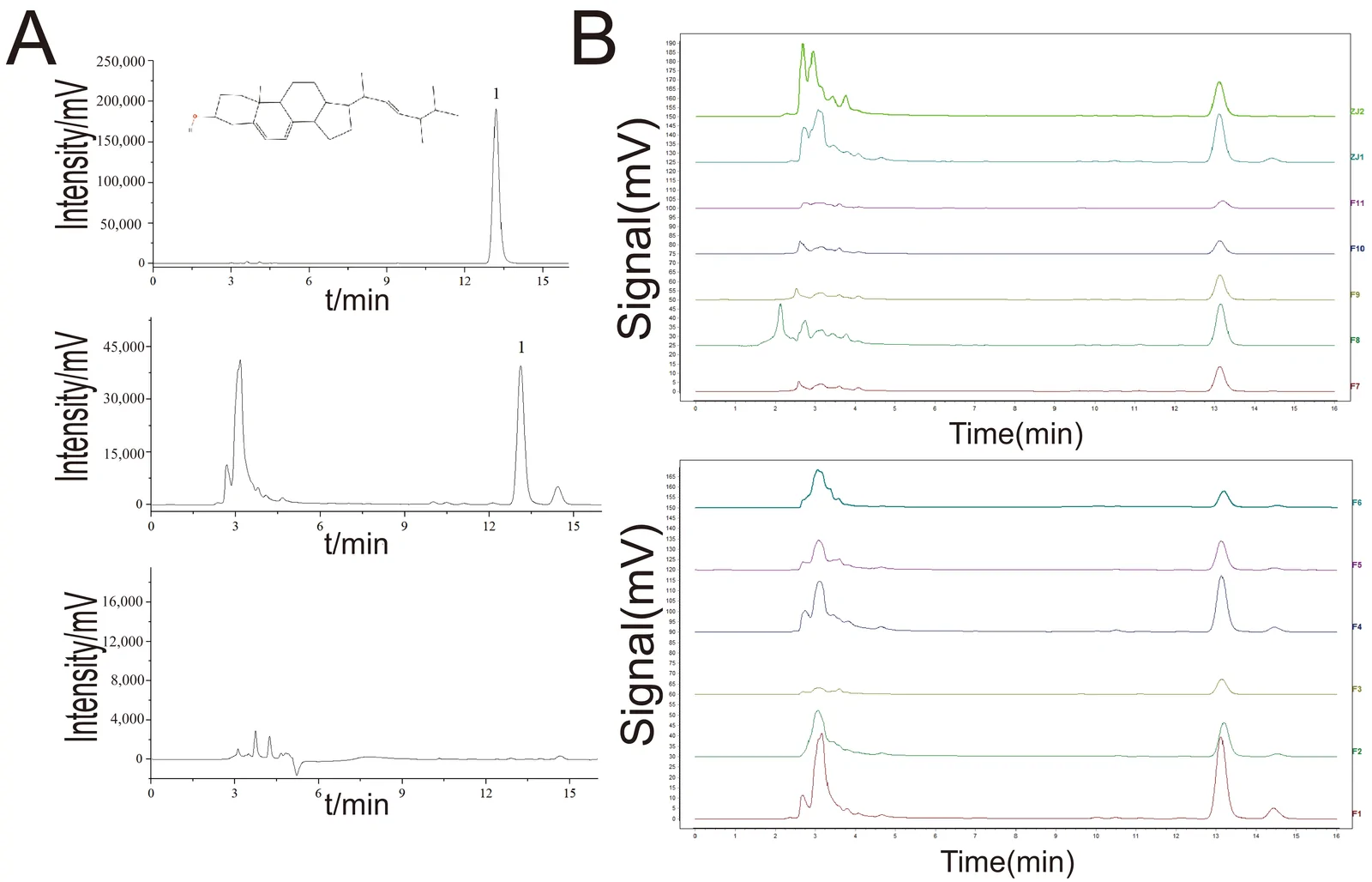
Determination graph of ergosterol content. (**A**) Exclusive chromatogram of ergosterol in red yeast rice samples from different sources. (**B**) Chromatogram of ergosterol in red yeast rice samples from different sources. 1 represents ergosterol. The curves in different colors represent the test results of different samples.

**Figure 4:**
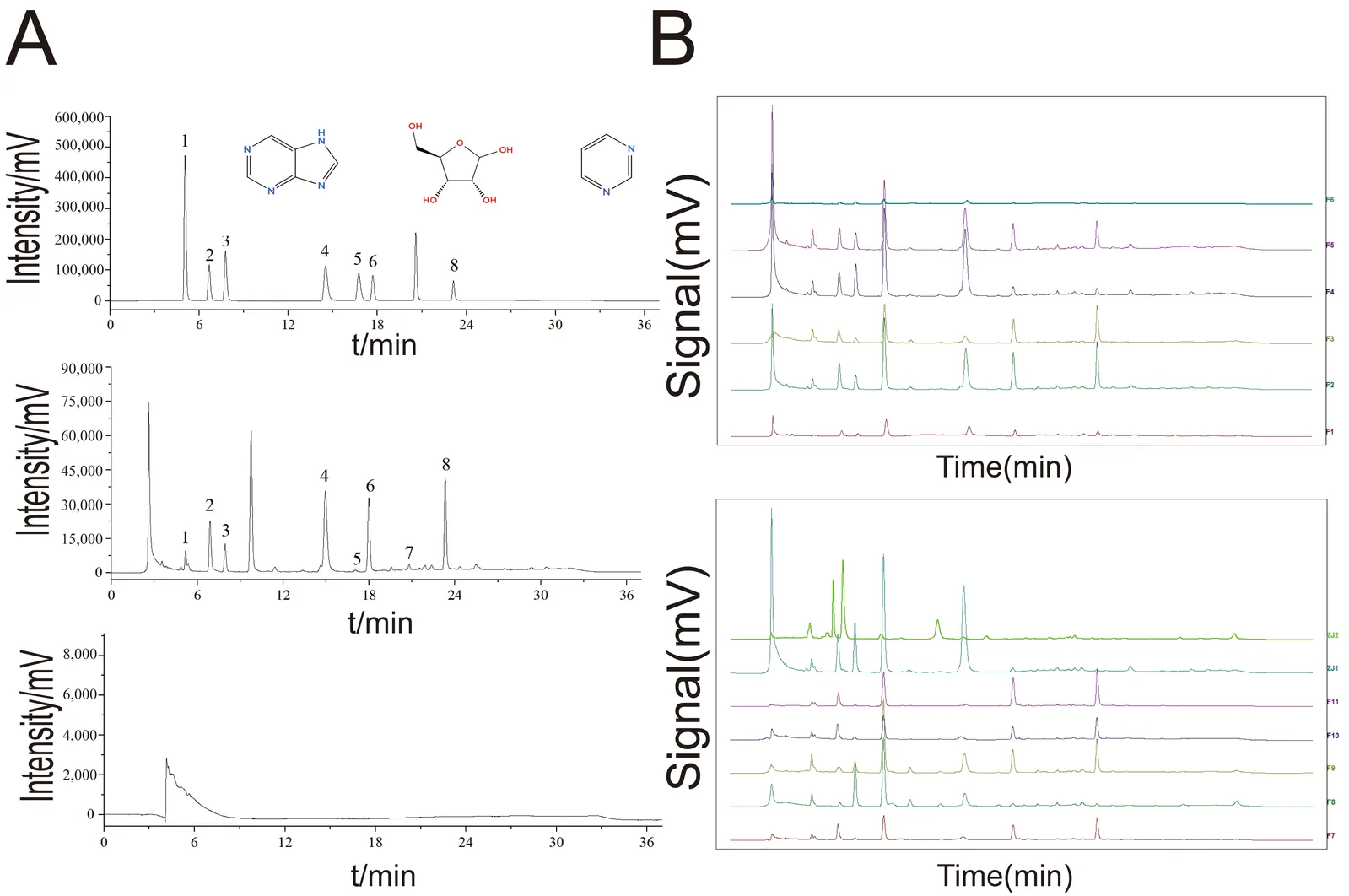
Determination diagram of nucleoside component content. (**A**) Exclusive chromatogram of nucleoside components in red yeast rice samples from different sources. (**B**) Chromatogram of nucleoside components in red yeast rice samples from different sources. The peaks on the chromatograms for detected nucleoside components are as follows: (1) Uracil; (2) Cytidine; (3) Hypoxanthine; (4) Adenine; (5) Inosine; (6) Guanosine; (7) Thymidine; (8) Adenosine. The curves in different colors represent the test results of different samples.

**Figure 5:**
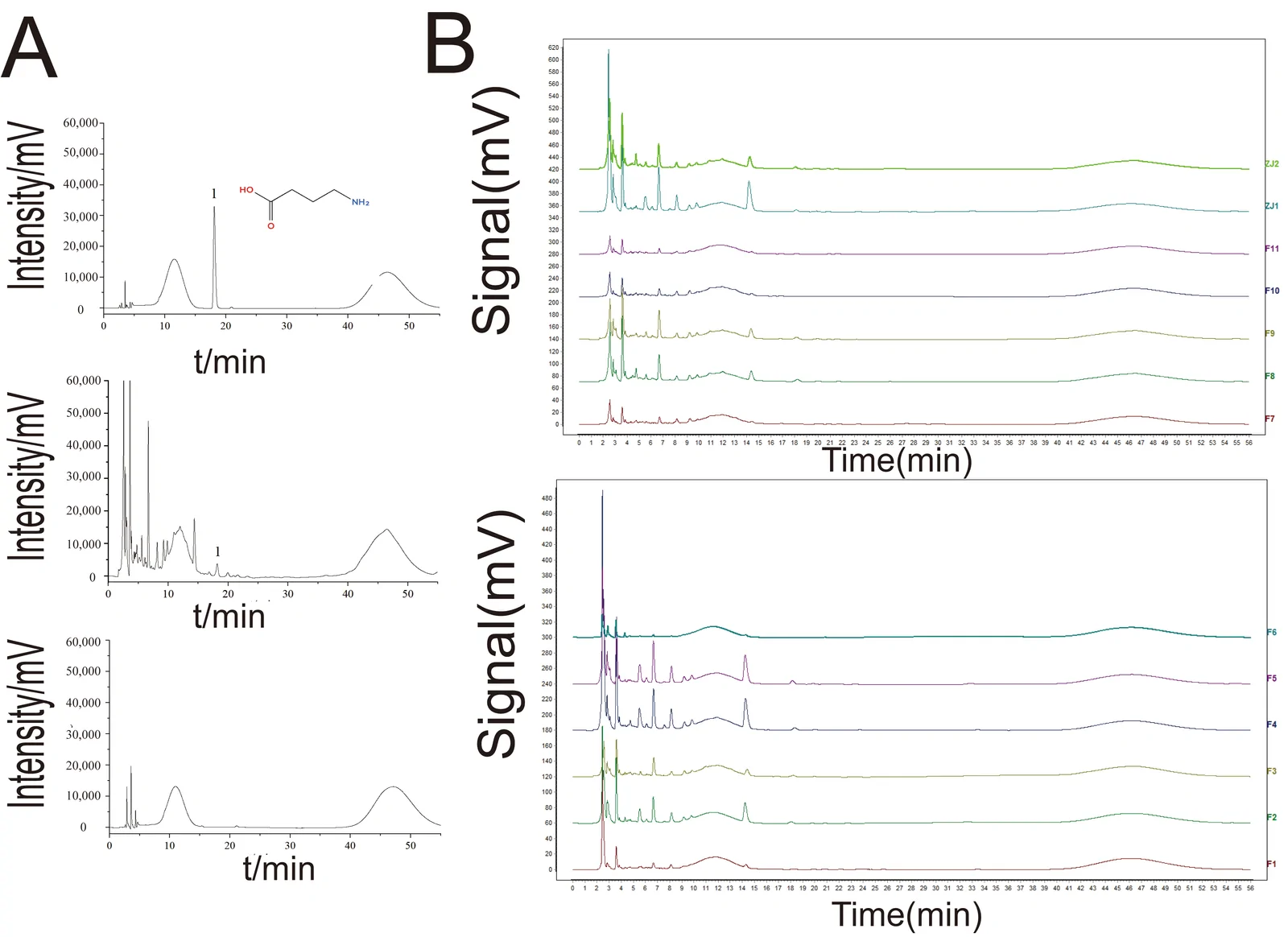
Determination graph of GABA content. (**A**) Exclusive chromatogram of GABA in red yeast rice samples from different sources. (**B**) Chromatogram of GABA-related components in red yeast rice samples from different sources. 1 represents GABA. The curves in different colors represent the test results of different samples.

**Figure 6:**
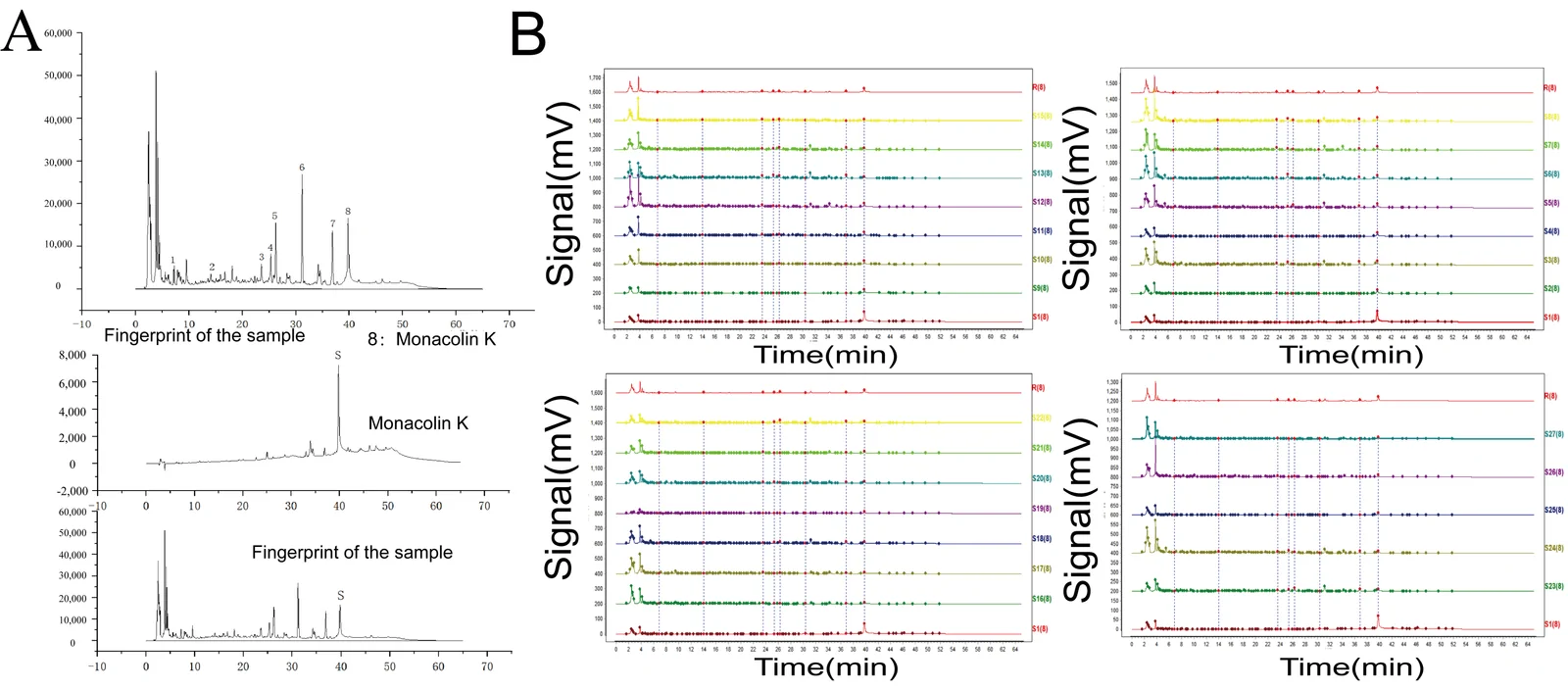
Establishment and analysis of fingerprints of red yeast rice samples from different sources. (**A**) Overlaid chromatographic peaks of the fingerprint spectrum for red yeast rice samples. Peak 8 and peak S represent Monacolin K. Peaks 1–7 are representative fingerprint peaks generated by the Similarity Evaluation System for Chromatographic Fingerprints of TCMs and do not correspond to specific chemical compounds. (**B**) Similarity evaluation plot for red yeast rice samples.

**Figure 7:**
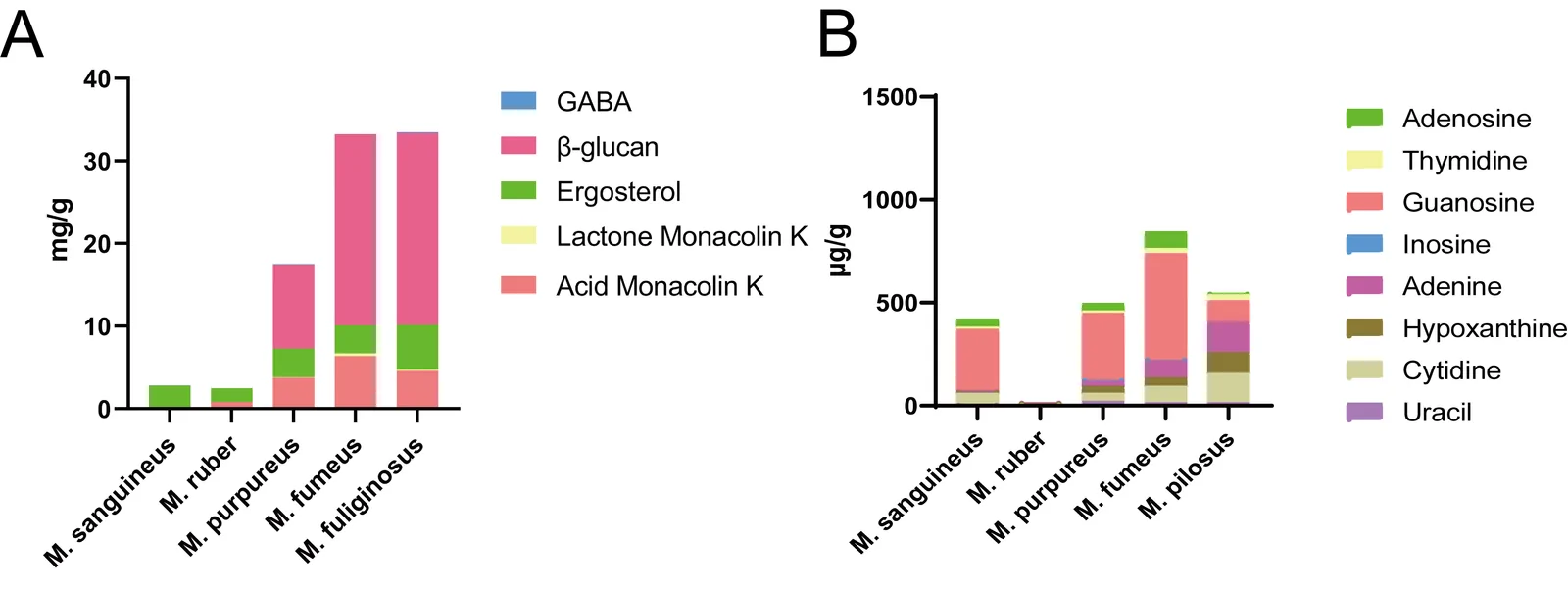
Comparison of contents of active components and nucleosides among various *Monascus* species. (**A**) Comparison of five key active components among five *Monascus* species; (**B**) comparison of eight nucleosides among five *Monascus* species.

**Table 1:** Species identification of isolated red yeast strains.

Sample ID	Species Identification	GenBank Accession Numbers	Similarity	Origin
F1	*Monascus purpureus*	MT584824.1	99.25%	Dingyuan Hongqu Co., Ltd., Ningde, China
F2	*Monascus fumeus*	GQ121015.1	99.80%	Yuanheng Quye Co., Ltd., Ningde, China
F3	*Monascus purpureus*	MZ297277.1	99.63%	Chengjiu Hongqu Co., Ltd., Ningde, China
F4	*Monascus fuliginosus*	HM188443.1	97.02%	Shiji Liangyou Biotechnology Co., Ltd., Ningde, China
F5	*Monascus purpureus*	MT558931.1	99.22%	Dingyuan Hongqu Co., Ltd., Ningde, China
F6	*Monascus ruber*	MT316354.1	100.00%	Diyuan Hongqu Factory, Ningde, China
F7	*Monascus sanguineus*	MZ297245.1	98.91%	Jiayuan Hongqu Rice Processing Factory, Ningde, China
F8	*Monascus purpureus*	MZ297277.1	99.44%	Pinghu Distillery Factory, Ningde, China
F9	*Monascus purpureus*	MZ297277.1	99.80%	Xingwang Hongqu Co., Ltd., Ningde, China
F10	*Monascus purpureus*	MT361827.1	95.16%	Shunjiang Hongqu Co., Ltd., Ningde, China
F11	*Monascus purpureus*	MZ297277.1	99.82%	Lifei Quye Co., Ltd., Ningde, China
ZJ1	*Monascus purpureus*	MT361827.1	99.08%	market, Huzhou, China
ZJ2	*Monascus purpureus*	MZ297277.1	96.92%	market, Huzhou, China

## Data Availability

The study includes the original findings, which are incorporated in the Section 2. For additional inquiries, please contact the corresponding authors.

## Supplementary Materials

The following supporting information can be downloaded at: https://www.mdpi.com/article/10.3390/jof12100734/s1, Table S1: the information of the additional 14 Red yeast rice samples; Table S2: The concentration of citrinin of detected samples; Table S3: The numerical values of analyzed compounds of Red yeast samples.
