# Supplementary material for: From Strain to Substance: A Novel Approach to Red Yeast Rice Quality Assessment and Chromatographic Fingerprinting
Source: J Fungi (Basel). 2026 Oct 1;12(10):734. doi: 10.3390/jof12100734 (PMC13705667; doi:10.3390/jof12100734)
Supplement: Supplementary file 1 [file jof-12-00734-s001.zip › Supplementary materials for table S1 and S2.pdf]

# Supplementary materials

**Table S1** the information of the additional 14 Red yeast rice samples

| Sample ID | Location | Origin |
|---|---|---|
| F14 | Zhejiang province, China | Market |
| F15 | Anhui province, China | Market |
| F16 | Anhui province, China | Market |
| F17 | Anhui province, China | Market |
| F18 | Fujian province, China | Market |
| F19 | Fujian province, China | Market |
| F20 | Fujian province, China | Market |
| F21 | Fujian province, China | Market |
| F22 | Zhejiang province, China | Tongjuntang Pharmaceutical Co., Ltd. |
| F23 | Hubei province, China | Wuhan Jiacheng Biological Products Co., Ltd. |
| F24 | Anhui province, China | Anhui Dechang Pharmaceutical Co., Ltd. |
| F25 | Anhui province, China | Anhui Dechang Pharmaceutical Co., Ltd. |
| F26 | Anhui province, China | Anhui Dechang Pharmaceutical Co., Ltd. |
| F27 | Anhui province, China | Market |

**Table S2** The concentration of citrinin of detected samples

| Sample ID | Peak area | Concentration ng/g |
|---|---|---|
| F1 | 0 | 0 |
| F2 | 0 | 0 |
| F3 | 78 | 140.343 |
| F4 | 0 | 0 |
| F5 | 318 | 604.922 |
| F6 | 0 | 0 |
| F7 | 0 | 0 |
| F8 | 2 | 0 |
| F9 | 9 | 0 |
| F10 | 0 | 0 |
| F11 | 6438 | 12451.69 |
| ZJ1 | 0 | 0 |
| ZJ2 | 0 | 0 |
